# The effects of decentralized financing and funding levels on the breadth of services and structural quality to provide those services in primary health facilities in Nigeria

**DOI:** 10.1186/s12913-025-12512-3

**Published:** 2025-03-18

**Authors:** Brittany Hagedorn, Jeremy Cooper, Benjamin Loevinsohn, Valentina Martufi

**Affiliations:** 1 Institute for Disease Modeling, Gates Foundation, Seattle, USA; 2 Gavi The Vaccine Alliance, Geneva, Switzerland; 3 Centro de Integração de Dados e Conhecimentos para Saúde (CIDACS), FIOCRUZ, Salvador, Brazil

**Keywords:** Nigeria, PHC, Structural quality, Facility financing, Services

## Abstract

**Background:**

To improve service delivery of Nigeria’s primary health care (PHC) system, the government tested two approaches for facility-level financing: performance-based financing (PBF) and decentralized facility financing (DFF). Facilities also had increased autonomy, supervision, and community oversight. We examine how the intervention approach and funding level affected breadth of services and structural quality.

**Methods:**

We use health facility surveys previously collected in 2014 and 2017, covering three years of implementation, in which districts were randomly assigned PBF or DFF and compared to matched districts in control states. We use log-linear regressions and non-parametric statistics to estimate the effect size of the financing approach and level of funding per capita.

**Results:**

Service availability was highest in PBF facilities, while DFF also outperformed control on most measures. Results showed that structural readiness and service offerings both increased with more funding, especially under DFF. DFF and PBF facilities were better equipped to provide services that they claimed to offer, which was not the case for controls. Overall, PBF outperformed DFF, partially explained by funding levels. The rate of offering complimentary services followed a pattern of easiest-to-hardest to deliver.

**Conclusion:**

PBF and DFF both improved the breadth and structural quality of services, although DFF performance was more sensitive to funding levels. Improvements were observed at relatively low levels of funding, but larger investments were associated with better performance. Most DFF facilities exceeded the performance of higher-funded controls, implying that funding was more valuable in the context of autonomy, increased supervision, and community oversight.

**Supplementary Information:**

The online version contains supplementary material available at 10.1186/s12913-025-12512-3.

## Introduction

Strengthening the primary health care (PHC) system in Nigeria is critical to improving the health of the population, but despite many efforts, the large PHC system, comprising more than 30,000 primary health care centers (PHCs) has struggled to perform [1–3]. 

To improve the quality of PHC services, the Nigerian Government launched the Nigeria State Health Investment Project (NSHIP), with a credit from the World Bank [4]. NSHIP used two financing approaches: performance-based financing (PBF) and decentralized facility financing (DFF), both of which provided facilities with supplemental direct financing (funds deposited into the bank accounts of the facilities), supportive supervision, community engagement, and autonomy to spend resources [4]. By design, DFF facilities received half of the level of financial resources as PBF facilities and were not allowed to spend on financial rewards for staff [5]. The details of PBF and DFF and their impact on population coverage outcomes is described elsewhere [6]. 

The NSHIP was piloted in three states: Adamawa, Nasarawa, and Ondo. Local Government Areas (LGAs) were randomly assigned to either the PBF or DFF scheme. LGAs from three control states were selected by matching socio-demographic characteristics to those in the intervention states. Aside from ethno-religious and economic traits, the three intervention states differed in terms of implementation quality [7], as well as important contextual factors (especially the conflict in Adamawa State) [8] that affected implementation.

To provide high quality care, PHCs need human resources, infrastructure (such as water and electricity), equipment, medicines, and supplies. These were in short supply in some facilities at the start of NSHIP [9]. Facilities also need to have adequate opening hours and limit financial barriers to care to meet the needs of the community [10–12]. 

There are many definitions of ‘quality of care’. In 1988, Donabedian proposed a framework that included structure, process, and outcomes [13] and this model remains a common paradigm. More recent approaches have taken a more prescriptive approach for specific health domains [14, 15]. For example, in some geographies such as India, structural quality standards that define a minimum set of requirements (e.g., equipment availability) are used for health care facility certification and incentive programs [16]. 

Previous studies have shown that incremental funding, such as that provided under PBF and DFF, can sometimes improve structural quality, process of care, and increases in service utilization [17–20]. However, the evidence for effects on health outcomes is mixed and inequalities can persist [18, 21–24]. For example, increasing skilled birth attendance does not guarantee a reduction in maternal mortality, as was seen in Liberia and Ethiopia [25, 26]. Taken in aggregate, these findings suggest that structural quality is necessary but not sufficient to increase utilization and improve quality.

NSHIP aimed to improve both service volumes and quality of care by providing direct financing, autonomy, and enhanced supervision to health facilities. The program showed increases in structural quality and service coverage in both DFF and PBF facilities, relative to the control arm [6, 27]. Specifically, both DFF and PBF facilities achieved higher rates of population level coverage of immunization, modern contraception, and institutional deliveries, but did not increase antenatal care or bed net use. However, few studies have examined whether or how facilities prioritize the services they provide or their investments in structural quality. We explore this prioritization process as health facilities obtain higher levels of funding and gain greater autonomy over spending decisions.

## Materials & methods

We examine the association between NSHIP study arms, self-reported services offered, and facility readiness to provide those services. We layer together four analyses which examine different aspects of these variable and combine them to provide a comprehensive picture of the changes that took place during the NSHIP.

We only include PHCs and exclude hospitals. The term “study arm” differentiates the three groups of facilities: control, DFF, and PBF. Our data comes from two independently conducted health facility surveys that were carried out in the original six states (three control, three intervention). The first survey was conducted in early 2014 when NSHIP implementation was just beginning and serves as a baseline. The follow-on survey was carried out late in 2017, more than three years after NSHIP began [28, 29]. For simplicity, we refer to these surveys as ‘baseline’ and ‘endline’ while recognizing that the program extended beyond that timeframe.

We utilize the baseline and endline data together where possible to examine the effects of NSHIP on the outcomes of interest. However, this is not always possible and we have indicated where we are only using the endline data for analysis. For example, facility revenues and detailed services lists were only collected at the endline, so some regressions are limited in scope.

The facility surveys were generally complete, but where there was missing data, we have excluded incomplete observations and reported the sample sizes to reflect this.

To avoid bias, we were as inclusive as possible in this analysis. First, we include all services which were addressed in sufficient depth in the facility surveys and for which data was complete, with the aim of capturing as much of the national government’s minimum service package as possible. Second, we defer to the Nigerian government’s standards for PHCs as a starting point for defining structural quality, including all relevant survey questions matched to their minimum standards wherever possible [30]. 

### Breadth of services provided

We start by looking at the differences between the control and intervention facility services in the 2017 endline survey which includes 980 PHCs and ask whether NSHIP facilities provide a broader set of services. Previous work has shown that intervention facilities increased their service volumes so we do not reexamine that here [4, 8]. 

We examine both aggregate measures of offered services and packages of related services (e.g., eight obstetric-related offerings), based on having sensible bundled packages of care. This resulted in nine packages, four of which were aggregates: services offered (of 46 possible to offer), laboratory diagnostics available (of 18), point-of-care diagnostics in stock (of 6), general drugs in stock (of 29), and five of which were service breadth measures for: family planning (of 7), pregnancy (of 8), childhood immunizations (of 15), malaria (of 3), and HIV plus STI (of 7). Details are in Additional File 1, Table A1.1.

We use the non-parametric Mann Whitney U test to assess whether the means between study arms differ; we do so because the outcome measures are expressed as percentage (bounded from 0 to 100%), and so normality is not assured.

### Impact of funding

We then examine the effect of facility funding levels on service offerings, on the hypothesis that facilities with greater resources may choose to provide a more comprehensive package of services (e.g., offering both diagnosis and treatment, not only referral). Because of the study design, this requires us to disentangle the effect size of study participation, incentive structure, and funding level. We use a regression approach with the dependent variables being the same aggregate measures listed in Additional File 1, Table A1.1, as used in the previous analysis. The explanatory variables include state, study arm, facility-reported total revenues per capita (based on estimated catchment populations), and the interaction term between revenue and study arm. Revenues were reported by the facilities for calendar year 2016, by source (including DFF/PBF, user fees and charges, government-provided funds, and donations), and we totaled these for use in the regression. Adjusted R-squared and variable coefficients are reported in the results. An examination of facilities with incomplete revenue data (Table A1.2) and discussion of the implications for potential bias are in Additional File 1.

### Prioritization of services

We hypothesize that facilities may have chosen to add ‘easier’ services first and then expand into more complex service offerings if they had more resources. Having only two time points (baseline and endline), it is not possible to directly evaluate this. Instead, we look at family planning and pregnancy-related services and examine whether NSHIP facilities exhibit the pattern that would emerge in the endline survey if this hypothesis were true.

For family planning, this cascade of increasing complexity would be: condoms; short-acting methods (pills and injections); long-acting methods (implants and IUDs); and sterilization (male and female). For pregnancy services, this cascade would be: antenatal and postnatal care; low-intervention deliveries (spontaneous, assisted, and home delivery); and more complex deliveries (low birth weight and cesarean section).

### Structural quality and readiness to provide services

Ideally when offering a service, a facility should be well-prepared to provide high quality care, commonly referred to as structural quality. Not all facilities offer all potential services, so we can examine whether study participation affected structural quality for facilities offering a service.

Facilities self-reported their service offerings and we assess their structural quality for family planning, antenatal care, spontaneous childbirth, routine childhood immunization, and diagnosis and treatment for malaria, tuberculosis, HIV, and other STIs. We consider relevant facility infrastructure, guidelines, patient registers, equipment, drugs, and supplies (e.g., malaria diagnosis relies on RDTs) and exclude items that are not tied directly to one service (e.g., thermometers). Details are in Additional File 1, Table A1.3.

In theory, a facility that claims to offer a service should have more of the structural components in place to provide that service. To assess whether NSHIP affected this dynamic, we compare four sub-groups of facilities: those offering (or not) a service and whether a facility was an NSHIP intervention or control facility. Facilities were not asked about detailed service offerings in the baseline survey, so we only have information on a few general services (e.g., “does this facility provide immunization services”. In the endline survey, a comprehensive set of services was included (e.g., “does this facility provide IUDs?”). Because of the much greater resolution of information available we rely only on the endline, comparing the control with intervention facilities. This limits the interpretation of this part of the analysis to an associational, rather than causal, conclusion because states were selected to be representative but were not randomized.

To assess this relationship, we calculated a structural quality score (SQS) for each service by facility, totaling the number of items listed in Additional File 1, Table A1.3 that were in place on the day of the survey. We first conduct a Mann-Whitney U-test of means between the relevant pairs of facility groups. If the NSHIP program affected facility readiness to provide specific services, we would expect to see that a) intervention facilities that do offer a service are more prepared than those that do not and b) intervention facilities that offer a service are more prepared than control facilities with the same service offering.

To separate out the effect of study arm participation alone from its effect on facility investments in readiness, we then fit a linear regression to test whether intervention facilities that offered a service were better prepared relative to control facilities that offered the same service. The regression takes the following form where *i* is the service area and *j* in the facility. The coefficient of interest is on the interaction between Study Arm and Service Offered.

$$\begin{aligned} {SQS}_{i}=&\:{Study\:Arm}_{j}+\:{State}_{j}+{Service\:Offered}_{i,j}\\&+\:{Study\:Arm}_{j}+\:{Study\:Arm}_{j}*{Service\:offered}_{i,j} \end{aligned}$$

## Results

For this analysis, we used the facility surveys from the NSHIP project in Nigeria. Intervention states were purposively selected based on baseline performance and their willingness to participate. Control states were selected to have similar baseline characteristics, resulting in a quasi-experimental design. Within the intervention states, LGAs were randomly assigned to DFF or PBF study arms. This process was described in the “Study Methodology” section under point 8, and the baseline facility performance was described in Tables 1 and 6 of the evaluation report [4] as well as in the publication of the key study findings [6]. For our secondary analysis, we used only primary healthcare facilities, resulting in a sample size of 258 control facilities, 325 DFF and 397 PBF.

### Breadth of offerings

We ran the Mann-Whitney U-test for means on the measures of breadth of services offered, as listed in Additional File 1, Table A1.1. We found the DFF and PBF facilities reported higher scores across all measures except for routine immunization, compared to control facilities, with thirty-two of thirty-six paired tests showing a statistically significant difference. However, there remained room for further improvement to achieve the maximum possible score. PBF facilities outperformed DFF facilities on every measure. Average scores and *p*-values for each paired test are reported in Table 1. The sample included 258 control facilities, 325 DFF facilities, and 397 PBF facilities, which was the subset with completed surveys for these questions (% missing). Standard deviations and u-values are in Additional File 1, Table A1.4.

**Table 1:** Impact of NSHIP on breadth of services

Measure	Mean, Control	Mean, DFF	Mean, PBF	*p*-value, DFF versus Control	*p*-value, PBF versus Control	*p*-value, PBF versus DFF
# self-reported services offered, total (46)	20.7	20.7	25.6	0.12	< 0.001	< 0.001
# Lab diagnostics, last three months (18)	3.7	5.1	7.6	< 0.001	< 0.001	< 0.001
# Point-of-care diagnostics, available today (6)	1.8	3.0	3.9	< 0.001	< 0.001	< 0.001
# General drugs, available today (29)	7.8	12.5	16.6	< 0.001	< 0.001	< 0.001
# Family planning options offered (7)	2.9	3.0	4.0	0.86	< 0.001	< 0.001
# Pregnancy services (8)	3.4	3.5	4.4	0.04	< 0.001	< 0.001
# immunizations offered (9)	8.5	7.6	8.7	< 0.001	0.35	< 0.001
# malaria services (3)	1.5	1.8	2.1	< 0.001	< 0.001	0.002
# HIV + STI services (7)	1.3	1.5	2.4	0.08	< 0.001	< 0.001
Sample size	258	325	397	-	-	-

Average number of service offerings, calculated by study arm

*P*-values are derived from a Mann-Whitney test of means. Measure descriptions are in Table 1. Maximum possible scores (# of services) are indicated in parentheses in far-left column

*DFF* direct facility financing, *PBF* performance-based financing, *HIV* human immunodeficiency virus, *STI* sexually transmitted infections

### Impact of levels of funding

**Fig. 1:**
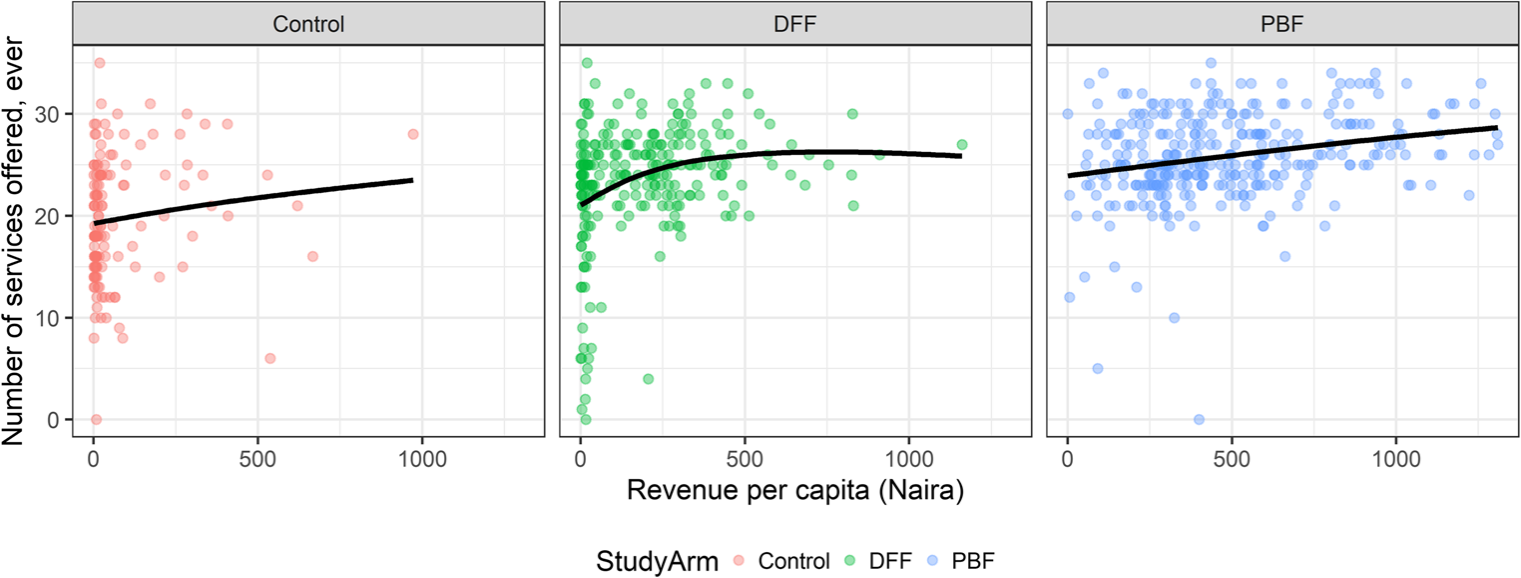
Association between revenues and service offerings. Facilities reported 2016 total revenues (from all sources) and the services that they offer, which are summarized and shown. Each point indicates the values for an individual facility. Revenue per capita was calculated as total reported revenues divided by the number of individuals reported to be in the facility’s catchment population. Lines shown are smoothed averages. DFF = direct facility financing. PBF = performance-based financing. 100 Naira ~ $ 0.27 USD

There are multiple potential reasons that the NSHIP facilities offered additional services to their communities. A qualitative review of the data suggested that incentives specific to the study arm and revenues available to facilities to expand services may be important drivers (Fig. 1). Revenues per capita were calculated based on facility-reported catchment populations, after removing erroneous values (e.g., zeroes and NAs) and outliers exceeding the 95th percentile. Sample size was 714 facilities with complete data. Control facilities had an average of 80 Naira (US$0.21) per capita in reported total 2016 revenues, DFF had 195 Naira (US$0.52) and PBF 484 Naira (US$1.30).

To disentangle possible drivers, we used bivariate regression analysis to assess the effect size of revenue and program participation on facilities’ structural quality (Table 2). Of the nine outcomes considered, seven had adjusted R-squared values above 0.20, with ‘number of general drugs in stock’ the highest at 0.34. At *p* *≤* 0.05, revenue was significant in all regressions and study arm was significant in seven of nine, which is substantially above what would be expected from random chance (fewer than one). The interaction term was never significant and was removed from the regression. Participation in the NSHIP intervention had the largest effect on predicting the number of total services offered and the number of general drugs available, which are also the measures with the highest possible total score. Of note, the two regressions for which study arm was not significant were the vertical programs of routine immunization and HIV.

Effect size for intervention and revenues on facility service offerings.

**Table 2:** Regression results for analyses estimating the effect size of intervention and available revenues on the number of service offerings provided by a PHC facility

Measure	Adj. *R* Sq.	Co-efficient Revenue	Co-efficient DFF	Co-efficient PBF	Max Possible	*N*
# Service offered, total	0.26	0.107 ***	0.122 ***	0.143 ***	46	714
# Lab diagnostics, last three months	0.28	0.106 ***	0.089 **	0.130 ***	18	714
# Point-of-care diagnostics, today	0.28	0.119 ***	0.318 ***	0.354 ***	6	714
# General drugs, today	0.34	0.091 ***	0.295 ***	0.347 ***	29	714
# Family planning options	0.25	0.081 **	0.209 ***	0.268 ***	7	714
# Pregnancy services	0.15	0.067 **	0.113 ***	0.156 ***	8	714
# immunizations offered	0.05	0.035 *	NA	NA	15	714
# malaria services	0.25	0.281 ***	0.422 ***	0.341 ***	3	714
# HIV + STI services	0.25	0.217 ***	NA	NA	7	714

Measure definitions are in Additional File 1, Table A1.1. # = number of items. Max possible = the number of services a facility could have offered. N = sample size. Revenue is total facility revenue per capita (1,000 N ~ 2.68 USD). Results from non-normalized outcomes (counts, rather than proportions) are in Additional File 1, Table A1.5

*DFF * direct facility financing, *PBF *performance-based financing, *HIV * human immunodeficiency virus, *STI* sexually transmitted infections, *NA * not significant

Statistical indicated by: *** is *p* < 0.001, ** is *p* < 0.01, and * is *p* < 0.05

### Prioritization of services

**Fig. 2:**
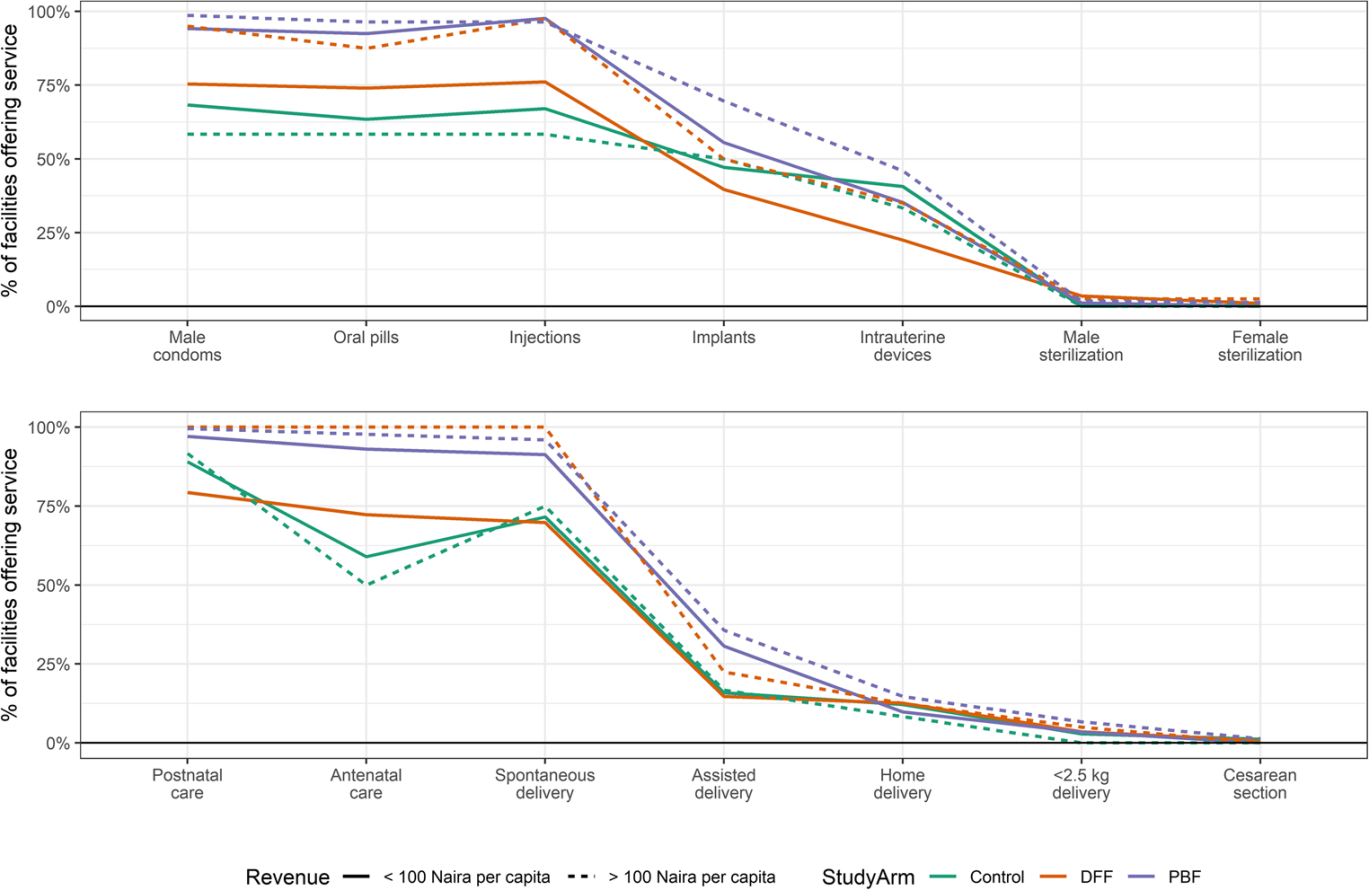
Percentage of facilities offering services, by study arm and level of revenues. Services are listed from lowest to highest complexity (left to right), based on required provider skill level, supplies, and equipment. Revenue per capita was calculated from reported total facility revenues in 2016, divided by total catchment population for the facility. DFF = direct facility financing. PBF = performance-based financing. 100 Naira ~ $ 0.27 USD

A greater proportion of facilities offer services that are simpler to provide, generally declining as services require greater skill and additional service readiness, for both family planning and pregnancy-related services. For example, more facilities offer contraceptive pills than implants. (Fig. 2) Grouping facilities by revenues (above/below $1.00), shows little differentiation for control or PBF facilities, but DFF with more revenues have higher rates of offering services. Further, PBF facilities have higher offering rates than DFF, which in turn have higher rates than control, even within the same revenue categories.

We present all services surveyed, although some are linked in practice (e.g., a facility that offers spontaneous delivery should be able to handle low-birth weight babies because these cannot always be anticipated).

### Structural quality and readiness to provide services

Performance on structural quality varied widely by study arm and service offering. PBF facilities generally had higher readiness than DFF, which in turn outperformed control facilities across most services. Further, facilities that offer a given service had higher levels of readiness to provide it than those that do not. There is an additional increase in readiness amongst those facilities that offer a service and were also in the intervention arm. For example, for antenatal care, DFF and PBF facilities combined had a 28.5% higher structural readiness score than control facilities offering the same service; this ranged from 5.1% (FP implants) to 71% (spontaneous childbirth). Mann-Whitney comparison of means tests confirm these observed differences, demonstrating that a) facilities that offered a given service consistently had higher service readiness than did facilities that did not offer the service and b) this is especially true for PBF/DFF facilities (Table 3). Facilities with incomplete reporting were dropped from the analysis, affecting an average of 3.5% of facilities.

**Table 3:** Average structural quality scores were calculated for facilities in each study arm, as defined in Additional file 1, Table A1.1

	Mean structural quality score					*p*-value		*N*		
Service	Control (all)	DFF (all)	PBF (all)	DFF + PBF (offer service)	Control (offer service)	Offer service vs. not (DFF + PBF)	DFF + PBF vs. control (offer service)	Control	DFF	PBF
FP capacity	1.98	2.80	3.43	3.32	2.49	< 0.001	< 0.001	251	304	396
FP oral	0.53	0.76	0.86	0.90	0.78	< 0.001	< 0.001	251	302	395
FP DMPA	0.45	0.67	0.77	0.78	0.64	< 0.001	< 0.001	251	302	395
FP implants	0.40	0.38	0.58	0.82	0.78	< 0.001	0.308	251	302	395
FP IUDs	3.36	3.69	4.36	4.92	4.23	< 0.001	< 0.001	251	301	395
ANC	6.67	8.20	9.18	9.15	7.12	< 0.001	< 0.001	254	304	396
Childbirth	18.99	29.18	36.24	35.25	20.65	< 0.001	< 0.001	247	299	394
Immunization	3.69	3.64	4.81	4.34	3.70	0.013	0.001	249	300	390
Malaria dx	1.05	1.64	1.76	1.77	1.30	< 0.001	< 0.001	248	301	395
Malaria treat	1.21	1.62	1.80	1.78	1.39	< 0.001	< 0.001	247	301	394
TB dx	0.45	0.70	0.90	2.27	1.59	< 0.001	0.011	250	303	394
TB treat	1.07	0.99	1.25	3.30	2.86	< 0.001	0.087	251	303	395
HIV dx	2.88	3.74	4.55	4.47	3.72	< 0.001	< 0.001	251	301	396
HIV treat	2.28	2.83	3.41	4.30	4.09	< 0.001	0.251	251	304	394
STIs	1.01	2.13	3.39	4.71	3.76	< 0.001	< 0.001	248	292	390

The Mann-Whitney tests difference in means between subgroups of the primary healthcare facilities and *p*-values indicate significance (or not) of those differences

*FP* family planning, *TB* tuberculosis, *HIV* human immunodeficiency virus, *STI* sexually transmitted infections, *DFF* direct facility financing, *PBF* performance-based financing, *Dx* diagnosis, *ANC* antenatal care

Structural quality scores and Mann-Whitney test results on difference in means.

**Fig. 3:**
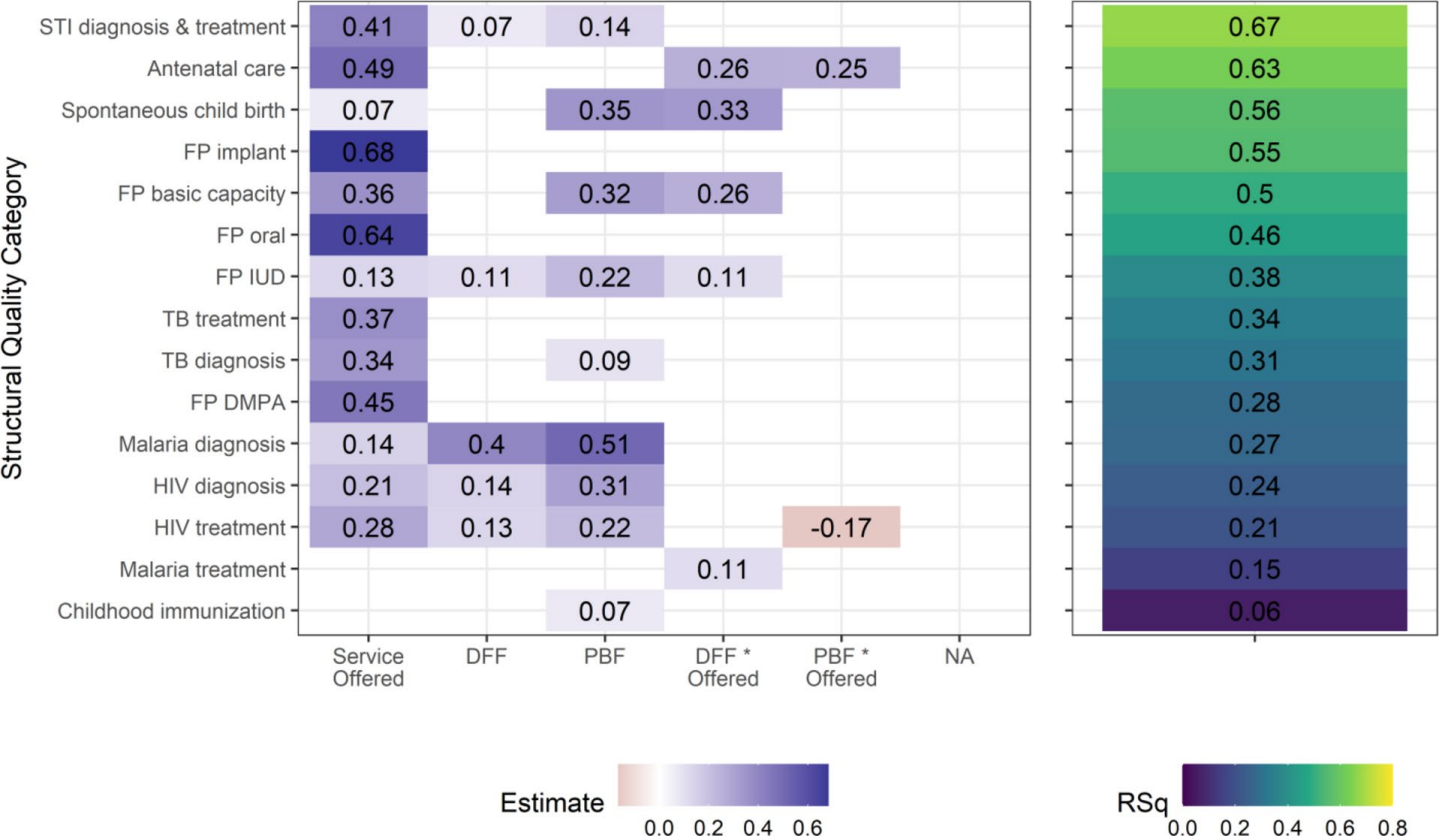
Results from linear regressions to predict structural quality. Each row represents a separate regression that was run, one for each outcome. The significant predictors are shown and their coefficients colored for interpretation, and the resulting R squared is also indicated. Left: Coefficient estimates are only shown for independent variables with a *p*-value < 0.05 (blank cells thus indicate non-significance). Text labels and shading indicate the value of the coefficient estimate. Regressions were normalized so that a score of 1.0 is a perfect score, as described in Table 2. “Study Arm * Offered” indicates the interaction term between these two independent variables. Right: Adjusted R-squared for the corresponding regressions. STI = sexually transmitted infection. FP = family planning. IUD = intrauterine device. TB = tuberculosis. DMPA = injectable contraceptive. HIV = human immunodeficiency virus

Using regression analysis to assess the impact that the NSHIP had on structural quality, we find that facilities that offer services are more likely to have the relevant structural components in place to provide overall quality care. In addition, being in the intervention arm had a positive effect on structural quality, with both the direct effect and the interaction term between study arm and offering a service being statistically significant *p* < 0.05. The interaction term was significant for DFF in five of fifteen regressions (antenatal care, spontaneous childbirth, family planning general capacity, IUD care, and malaria treatment), but significant for PBF in only two of fifteen regressions (one of which has the only negative coefficient estimate). Regression adjusted R-squared values were as high as 0.67 and above 0.50 for services including STI diagnosis and treatment, antenatal care, spontaneous childbirth, family planning capacity and implants (Fig. 3). Facilities with incomplete reporting were dropped from the analysis, affecting up to 5.2% of facilities in one regression; we tested for sample bias and did not observe any.

The largest and most consistent effect sizes found in these regressions were for the binary variable of ‘service offered or not’, indicating that facilities that do offer a service are more likely to have the equipment, supplies, etc. that they require. This is followed by being in the PBF arm of the study, and then there are some smaller effect sizes for DFF facilities, which had similar support systems but less funding than PBF facilities.

## Discussion

The objective of the NSHIP was to expand services and improve quality of care in PHC facilities in three states in Nigeria and we find that this was indeed the case. This aligns with previously published literature on the effects of financing on PHC that show that facility-level resources can positively impact performance [20, 21, 23]. 

By the time of the survey in 2017, NSHIP intervention facilities offered a more comprehensive set of services and products across most of the dimensions measured, with PBF providing even more comprehensive services than DFF (Table 1). For example, PBF facilities offered an average of five more services, intervention facilities offered 1–2 additional lab diagnostics, DFF facilities stocked five additional general-purpose drugs (such as antibiotics), and PBF more than doubled the number of drugs, all compared to control. Immunization was the one exception to this finding, likely because it was provided in almost all facilities regardless of study arm and is managed as a national program so is less likely to vary in response to financing or incentives.

We find that facility-level total revenues per capita are strongly associated with better performance, including offering more services, diagnostics, and products to their users. This is based on bivariate regression analyses (Table 2), in which revenue was significantly associated with outcome measures at the *p* < 0.01 level for all services except immunization. The effect size shown in the regression coefficients for revenue was up to 0.281 for malaria services, equivalent to a 2.8% increase per one hundred Naira per capita increase in revenues at the mean. While the coefficients are not always large, they can still be meaningful when it comes to overall impact on service availability, suggesting that even modest funding levels can expand access to healthcare. For example, looking at the total number of services offered, the coefficients are 0.11 for revenues, 0.12 for DFF, and 0.14 for PBF, which is equivalent to an increase of 4.9, 5.6, and 6.7 additional services per one hundred Naire per capita, respectively (See Additional File 1, Table A1.5).

Coefficients for DFF and PBF were also significant, which indicates that these facilities offered more services at a given level of revenue than control facilities did. From this, we conclude that having programmatic support (i.e., management and budget training plus supportive supervision) to support smart expenditure choices was important in achieving the observed improvements. In addition, the magnitude of the PBF coefficients are larger than those for DFF, even after accounting for higher PBF funding, implying that PBF increased services more overall, possibly due to the addition of an explicit incentive structure for certain behaviors. From these combined findings, we conclude that intervention facilities offered more comprehensive primary health care and were more prepared to provide high quality care, and that this was driven by both increased facility-level funding and the structured supportive supervision that they received.

The order in which services were added is also of interest, with the hypothesis that if financial resources were a barrier to offering care, then facilities would choose to offer simpler services that are less expensive to support. Indeed, the patterns of service offerings were generally consistent with this expectation, for example with more facilities offering oral contraceptives than implants (Fig. 2). Intervention facilities did not achieve fully comprehensive care, but they did outperform control across the board. The high rates of service offering amongst lower-revenue PBF facilities suggests that the explicit incentives to offer basic services were indeed effective in changing behavior. However, this conclusion is somewhat mitigated by the similar performance of DFF facilities with higher revenues, which did not receive direct incentives. This implies that the combination of adequate revenues with other aspects of the intervention package, such as supportive supervision and training, is enough to substantially increase service availability without the need for financial incentives.

In regressions to predict structural quality, the largest effect size is whether a service is offered or not (13 of 15 services), followed by being a PBF facility (9 of 15 services) and then DFF (5 of 15 services). The interaction terms are more frequently significant for DFF (5 of 15 services) than for PBF (Fig. 3). All of these coefficients are positive except for one (PBF HIV), which means that offering a service and being an NSHIP facility are positively associated with structural quality. Given that these outcome measures represent fairly complicated lists of equipment, staffing, and facility infrastructure, the positive response to NSHIP implies it may be due to the improved management quality that was supported by the intervention, especially early on in the PBF facilities.

In combination, PBF facilities have more structural quality overall, but DFF facilities have more frequent interaction effects. This may be partially attributable to the difference in explicit incentives between the study arms. The observed differences between DFF and PBF suggests that PBF facilities used their funding to increase overall facility readiness in accordance with the quarterly supervision checklist, while DFF facilities, which received half of the level of funding as PBF, had to be more careful in limiting their spending to only items on their top priorities. This combination of observations implies that policy makers with explicit goals of increasing access to higher-complexity services (e.g., IUDs or c-sections) will need to provide both adequate funding and explicit incentives to do so.

This study has several limitations. Primary is the risk that there may be unobserved confounders that affect the results. This is particularly a concern for the control states, which could have had simultaneous policy or funding changes that affected their applicability as comparators. Additionally, we cannot account for changes in the vertical disease programs that may have happened in parallel. We note that the household survey showed that control and intervention households had different baselines on some indicators; however, given the pattern in the differences, this suggests that any bias results in an underestimation of effect size for DFF/PBF. The second limitation is that we use the survey respondent’s statement that a service is (or not) offered in their facility as an accurate representation, although the response is not corroborated with documentation. This may introduce noise into the data, reducing the likelihood that we would detect a true underlying relationship. For example, immunization was “offered” in nearly all facilities at baseline, so even though stock levels increased during the study period, this was not reflected in our definition of “service offering”. Third, while we recognize its importance, we do not look at process quality due to lack of data availability. This presents an opportunity for future work, potentially leveraging data from similar financing studies, to explore an expanded definition of quality including perceptions and user experience [31]. Last, we acknowledge that reported performance on a checklist is not the same as strong facility operations and there is the risk that facilities were “performing out” rather than making fundamental improvements, which has been documented elsewhere [32, 33]. 

## Conclusions

In summary, we conclude that increased facility-level funding and programmatic support, including strong supervision, drove an expansion of service offerings and improved facility readiness to provide the services that they offered. Policy makers and donors should consider providing incremental funding directly to facilities, paired with management support. PBF facilities generally performed better than DFF, which was mostly attributable to their higher levels of funding, rather than explicit incentives. In response to having fewer resources, DFF facilities were more targeted in their approach to improvement and service expansion. When deciding how much funding to provide to facilities, decision makers should keep in mind this “targeted” behavior and recognize that without adequate resources, facilities are unlikely to be able to offer more complex services (e.g., IUDs), and either set expectations appropriately or provide explicit incentives for their priority services. We note that immunization, and to a lesser extent HIV, were often exceptions to the general patterns observed across the analyses; this may be attributable to the centralized, donor-driven management systems that provide free products and non-local decision making, leading NSHIP to have a limited impact on their performance. Improvement in these areas will require different kinds of interventions than those present in NSHIP and should be managed accordingly.

## Supplementary Information

Supplementary Material 1.

## Data Availability

The data used in this study are available from the World Bank’s Microdata Library at https://microdata.worldbank.org, reference number NGA_2017_HRBFIE-EL_v01_M.

## References

[1] Aregbeshola BS, Khan SM. Primary health care in Nigeria: 24 years after Olikoye Ransome-Kuti’s leadership. Front Public Health. 2017;5:48. 10.3389/fpubh.2017.00048. 28349050 10.3389/fpubh.2017.00048 PMC5346888

[2] Mabuchi S, Alonge O, Tsugawa Y, Bennett S. An investigation of the relationship between the performance and management practices of health facilities under a performance-based financing scheme in Nigeria. Health Policy Plan. 2022;37. 10.1093/heapol/czac040. 10.1093/heapol/czac040 35579285

[3] 36 States And The FCT To Share $1.5m FG Fund For Primary Healthcare. Lagos (Nigeria): Information Nigeria; 2016. https://www.informationng.com/2016/07/36-states-and-the-fct-to-share-1-5m-fg-fund-for-primary-healthcare.html. Accessed 20 Jul 2023.

[4] Kandpal E, Loevinsohn B, Vermeersch C, Pradhan E, Khanna M, Conlon M, et al. Impact evaluation of Nigeria state health investment project. Washington (DC): The World Bank; 2018.

[5] National Primary Health Care Development Agency, Federal Ministry of Health, Ondo Nasarawa and Adamawa State Ministries of Health. Nigeria State Health Investment Project (NSHIP) Performance-Based Financing User Manual, annex 7 of the Project Implementation Manual. Ntl Prim Health Care Dev Agcy. Abuja (Nigeria); 2013.

[6] Khanna M, Loevinsohn B, Pradhan E, Fadeyibi O, McGee K, Odutolu O, Fritsche GB, Meribole E, Vermeersch CMJ, Kandpal E. Decentralized facility financing versus performance-based payments in primary health care: a large-scale randomized controlled trial in Nigeria. BMC Med. 2021;19(1):224. 10.1186/s12916-021-02092-4. 34544415 10.1186/s12916-021-02092-4 PMC8452448

[7] Odutolu O, Ihebuzor N, Tilley-Gyado R, Martufi V, Ajuluchukwu M, Olubajo O, Banigbe B, Fadeyibi O, Abdullhai R, Muhammad AJG. Putting institutions at the center of primary health care reforms: experience from implementation in three States in Nigeria. Health Syst Reform. 2016;2(4):290–301. 10.1080/23288604.2016.1234863. 31514721 10.1080/23288604.2016.1234863

[8] Dabang P, Hazzad A. Suicide bomber kills 50 in Nigeria in mosque attack. Yola (Nigeria): Reuters; 2017. https://www.reuters.com/article/uk-nigeria-security-idUKKBN1DL0TS. Accessed 20 Jul 2023.

[9] Kyari GV, Dogara M, Gimba SE. An analysis of facility assessment of primary health care in Nigeria: a micro-evidence from Kaduna State. Wukari Int Stud J. 2022;6(2):21–21.

[10] Dotse-Gborgbortsi W, Nilsen K, Ofosu A, Matthews Z, Tejedor-Garavito N, Wright J, Tatem AJ. Distance is a big problem: a geographic analysis of reported and modelled proximity to maternal health services in Ghana. BMC Pregnancy Childbirth. 2022;22(1):672. 10.1186/s12884-022-04998-0. 36045351 10.1186/s12884-022-04998-0 PMC9429654

[11] Kruk ME, Pate M, Mullan Z. Introducing the lancet global health commission on high-quality health systems in the SDG era. Lancet Glob Health. 2017;5(5):e480–1. 10.1016/S2214-109X(17)30101-8. 28302563 10.1016/S2214-109X(17)30101-8

[12] Phillips DE, Dieleman JL, Lim SS, Shearer J. Determinants of effective vaccine coverage in low and middle-income countries: a systematic review and interpretive synthesis. BMC Health Serv Res. 2017;17(1):681. 10.1186/s12913-017-2626-0. 28950899 10.1186/s12913-017-2626-0 PMC5615444

[13] Donabedian A. The quality of care: how can it be assessed? JAMA. 1988;260(12):1743–8. 10.1001/jama.260.12.1743. 3045356 10.1001/jama.260.12.1743

[14] McCauley H, Lowe K, Furtado N, Mangiaterra V, van den Broek N. What are the essential components of antenatal care? A systematic review of the literature and development of signal functions to guide monitoring and evaluation. BJOG Int J Obstet Gynaecol. 2022;129(6):855–67. 10.1111/1471-0528.17029. 10.1111/1471-0528.17029 34839568

[15] Standards for improving the quality of care for small and sick newborns in health facilities. Geneva (Switzerland): The World Health Organization. 2020. ISBN: 978 92 4 001076 5.

[16] National Health Mission of India. National Quality Assurance Standards for Public Health Facilities 2020. New Delhi (India): Ministry of Health and Family Welfare, Government of India; 2020.

[17] Abdallah HA, Kapologwe NA, Kibusi SM. Routine childhood immunization service readiness and uptake in the selected border district councils of Tanzania: a cross-sectional study. Preprint: 10.21203/rs.3.rs-2219517/v1.

[18] Das A, Gopalan SS, Chandramohan D. Effect of pay for performance to improve quality of maternal and child care in low- and middle-income countries: a systematic review. BMC Public Health. 2016;16:321. 10.1186/s12889-016-2982-4. 27074711 10.1186/s12889-016-2982-4 PMC4831162

[19] Kambala C, Lohmann J, Mazalale J, Brenner S, Sarker M, Muula AS, et al. Perceptions of quality across the maternal care continuum in the context of a health financing intervention: evidence from a mixed methods study in rural Malawi. BMC Health Serv Res. 2017;17(1):392. 10.1186/s12913-017-2329-6. 28595576 10.1186/s12913-017-2329-6 PMC5465597

[20] Ruhago GM, John MB, Ngalesoni FN, Msasi D, Kapologwe N, Kengia JT, et al. Understanding the implication of direct health facility financing on health commodities availability in Tanzania. PLOS Glob Public Health. 2023;3(5):e0001867. 10.1371/journal.pgph.0001867. 37155608 10.1371/journal.pgph.0001867 PMC10166559

[21] De Walque D, Kandpal E, Wagstaff A, Friedman J, Neelsen S, Piatti-Fünfkirchen M, et al. Improving effective coverage in health: do financial incentives work? Washington (DC): The World Bank; 2022. 10.1596/978-1-4648-1825-7.

[22] Riese S. Associations of maternal health care structure and process quality with outcomes in Malawi. Baltimore (Maryland): Johns Hopkins University; 2019.

[23] Gage A, Bauhoff S. The effects of performance-based financing on neonatal health outcomes in Burundi, Lesotho, Senegal, Zambia and Zimbabwe. Health Policy Plan. 2021;36(3):332–40. 10.1093/heapol/czaa191. 33491082 10.1093/heapol/czaa191 PMC8058947

[24] Haemmerli M, Powell-Jackson T, Goodman C, Thabrany H, Wiseman V. Poor quality for the poor? A study of inequalities in service readiness and provider knowledge in Indonesian primary health care facilities. Int J Equity Health. 2021;20(1):239. 10.1186/s12939-021-01577-1. 34736459 10.1186/s12939-021-01577-1 PMC8567576

[25] King J, Tarway-Twalla AK, Dennis M, Twalla MP, Konwloh PK, Wesseh CS, et al. Readiness of health facilities to provide safe childbirth in Liberia: a cross-sectional analysis of population surveys, facility censuses and facility birth records. BMC Pregnancy Childbirth. 2022;22(1):952. 10.1186/s12884-022-05301-x. 36539750 10.1186/s12884-022-05301-x PMC9764703

[26] Weldearegay HG, Kahsay AB, Medhanyie AA, Godefay H, Petrucka P. Quality of and barriers to routine childbirth care signal functions in primary level facilities of Tigray, Northern Ethiopia: mixed method study. PLoS ONE. 2020;15(6):e0234318. 10.1371/journal.pone.0234318. 32530944 10.1371/journal.pone.0234318 PMC7292403

[27] Sato R, Belel A. Effect of performance-based financing on health service delivery: a case study from Adamawa State, Nigeria. Int Health. 2020;13(2):122–9. 10.1093/inthealth/ihaa026. 10.1093/inthealth/ihaa026 PMC7902678 32530041

[28] Kandpal E. State health investment project: impact evaluation endline survey, 2017. The World Bank Development Data Group Microdata Catalog; 2017. https://microdata.worldbank.org/index.php/catalog/study/NGA_2017_HRBFIE-EL_v01_M. Accessed 20 Jul 2023.

[29] Martufi V, Garnvwa H, Muhammad M, Ajuluchuku M, Banigbe B, Alade M. The Nigeria State Health Investment Project (NSHIP) 2015 Annual Report. Abuja (Nigeria): National Primary Health Care Development Agency; 2016.

[30] National Primary Healthcare Development Agency. Minimum standards for primary health care in Nigeria. Abuja (Nigeria): Federal Government of Nigeria; 2012.

[31] Hanefeld J, Powell-Jackson T, Balabanova D. Understanding and measuring quality of care: dealing with complexity. Bull World Health Organ. 2017;95(5):368–74. 10.2471/BLT.16.179309. Epub 2017 Mar 20. PMID: 28479638; PMCID: PMC5418826. 28479638 10.2471/BLT.16.179309 PMC5418826

[32] Das P, Newton-Lewis T, Khalil K, Rajadhyaksha M, Nagpal P. How performance targets can ingrain a culture of ‘performing out’: an ethnography of two Indian primary healthcare facilities. Soc Sci Med. 2022;300:114489. 10.1016/j.socscimed.2021.114489. 34702616 10.1016/j.socscimed.2021.114489

[33] Newton-Lewis T, Munar W, Chanturidze T. Performance management in complex adaptive systems: a conceptual framework for health systems. BMJ Glob Health. 2021;6(7):e005582. 34326069 10.1136/bmjgh-2021-005582 PMC8323386

