# Supplementary material for: The effects of decentralized financing and funding levels on the breadth of services and structural quality to provide those services in primary health facilities in Nigeria
Source: BMC Health Serv Res. 2025 Mar 18;25:394. doi: 10.1186/s12913-025-12512-3 (PMC11916157; doi:10.1186/s12913-025-12512-3)
Supplement: Supplementary file 1 — Supplementary Material 1. [file 12913_2025_12512_MOESM1_ESM.docx]

**Additional File 1**

Table A1.1 Outcome measure designs. There were nine outcomes measures designed for this study, including both aggregate and breadth of offerings measures. These are described in detail below, along with information on which survey questions they include. Some measures have more components than others, depending on how many survey questions were relevant. All relevant components were included in the measure, and we created a measure for each service line that was asked about in the survey that had three or more component questions. Question references match those listed in the endline data survey instrument; they indicate the survey section, question number, and sub-question number where appropriate.

| **Type** | **Measure** | **Survey reference** |
| --- | --- | --- |
| Aggregate | Number of services offered, ever | Q 13.01, all services 1-46 |
|  | Number of laboratory diagnostics able to perform in last 3 months | Q10.08_test, all 1-18 |
|  | Number of point-of-care diagnostic tests in stock today | Q 11.20  50 = TB diagnostic kits  66 = malaria rapid diagnostic kits  67 = HIV test kit  68 = pregnancy testing kit  69 = rapid plasma reagin (RPR) test for syphilis  70 = urine testing kit |
|  | Number of general drugs in stock | Q 1120_drugs, all 1-29 |
| Breadth of offerings | Number of family planning options offered | Q 13.01  1 = contraceptive pill  2 = injection  3 = implant insertion  4 = male condoms  5 = IUD insertion  6 = female sterilization  7 = male sterilization |
|  | Number of pregnancy services offered | Q 13.01  8 = antenatal care  9 = spontaneous vaginal delivery  10 = cesarean section  11 = assisted delivery  12 = home delivery with skilled staff  13 = postnatal care  14 = low birth weight delivery  30 = Tetanus Toxoid for pregnancy |
|  | Number of routine childhood immunizations offered  Note: If a facility offers either pentavalent or both DPT and Hep B, this counts as equivalent. | Q 13.01  15 = Bacille Calmette Guerin (BCG)  16-18 = Pentavalent dose 1-3  19-21 = DPT dose 1-3  22-24 = Hep B dose 1-3  25-27 = Polio dose 1-3  28 = Measles dose 1  29 = Yellow fever |
|  | Number of malaria services offered | Q 13.01  34 = diagnosis of falciparum malaria  35 = diagnosis of non-falciparum  36 = treatment with ACT |
|  | Number of HIV + STI services offered | Q 13.01  40 = diagnosis, treatment, and counseling for syphilis  41 = diagnosis, treatment, and counseling for gonorrhea  42 = diagnosis, treatment, and counseling for chlamydia  43 = diagnosis, treatment, and counseling for other STDs  44 = voluntary counseling and testing (VCT) for HIV  45 = prevention of mother-to-child transmission of HIV  46 = antiretroviral treatment (ART) |

**Discussion of facilities that did not report revenues**

There were 146 facilities that did not report revenues in the facility survey. These are non-reported values (NA) in the survey and do not reflect true zeroes, which were sometimes documented for specific sources of revenues for other facilities in accordance with study protocols.

It was not possible to use interpolation to fill in these missing values because we do not find that these facilities were missing at random, so the methodological assumptions required for interpolation [Austin et al 2020] are violated. See service offering scores in table A1.2, which indicates that facilities that did not report revenues had statistically significantly lower service offerings.

This raises the question of whether the subset of facilities that did not report revenues (and were thus dropped from the analysis sample) may bias our results. We test this by examining the theory behind why these facilities did not report revenues. Mechanistically, we would expect that non-reporting is caused by poor documentation and thus non-availability of good quality data. This poor documentation in turn is likely caused by poor management practices at the facility level, which we would expect to also result in reduced facility offerings. If this theory is true, then the missing data would be expected to be consistent with our findings in the main paper – i.e., facilities with lower management scores should also have reduced service offerings and thus lower revenues.

We can partially test these theoretical relationships by examining whether the facilities that don’t report revenues have both lower management scores and facility offerings. In fact, this is what we find, (Table A1.2), suggesting that while the analysis in the manuscript is based on an imperfect dataset, the risk that the results are fundamentally flawed is low. In addition, we note that the only metric that does not show a significant difference between the facilities with and without revenue is question 6.05, indicating that these two groups of facilities had equivalent autonomy available to them to choose which services that they offered, reducing the risk of bias from missing data.

Table A1.2 Relative performance scores, comparing facilities that did or did not report revenues in the facility survey. Service offerings are defined consistently with table A1.1. Facility management scores are reported directly as defined in the facility survey, normalized to a scale of 0 to 1 where higher is better, in order to have consistent interpretation with service offerings indices.

|  | **Score** | **Mean, facilities that reported revenues** | **Mean, facilities that didn’t report revenue** | **p-value** |
| --- | --- | --- | --- | --- |
| Service offering | # of services offered, ever | 0.52 | 0.40 | <0.001 |
|  | # of laboratory diagnostics able to perform in last 3 months | 0.36 | 0.18 | <0.001 |
|  | # of point-of-care diagnostic tests in stock today | 0.57 | 0.31 | <0.001 |
|  | # of general drugs in stock | 0.50 | 0.27 | <0.001 |
|  | # of family planning options offered | 0.51 | 0.37 | <0.001 |
|  | Number of pregnancy services # | 0.51 | 0.38 | <0.001 |
|  | # of childhood immunizations offered | 0.57 | 0.48 | <0.001 |
|  | # of malaria services offered | 0.65 | 0.52 | <0.001 |
|  | # of HIV + STI services offered | 0.28 | 0.18 | <0.001 |
| Facility management | Q6.01: I am able to allocate my facility budget according to how it is needed. | 0.64 | 0.59 | <0.001 |
|  | Q6.05: I have choice over what services are provided in the facility. | 0.58 | 0.58 | 0.26 |
|  | Q2.11: Has a facility budget been developed for the current financial year? | 0.39 | 0.19 | <0.001 |
|  | Q2.12: Does the facility have business plan or activity plan developed for the current year? | 0.50 | 0.23 | <0.001 |
|  | Q2.19: Are priority health-related activities  identified for the current year? | 0.61 | 0.48 | <0.001 |

Table A1.3. Definition of structural quality by type of service. All questions listed here were included in calculating a facility’s readiness score. Survey reference is the specific question as defined in the endline facility survey conducted in 2017 by the NSHIP study. IEC/BCC = Information, education and communication/behavior change communication. IUD = intrauterine device. ANC = antenatal care. RDT = rapid diagnostic test. TB = tuberculosis. HIV = human immunodeficiency virus. AIDS = acquired immunodeficiency syndrome. CD4+ count = a test of white blood cell counts. VCT = voluntary counseling and testing. PMTCT = prevention of mother to child transmission of HIV. STI = sexually transmitted infection. RPR = rapid plasma reagin. BEmONC = basic emergency obstetric and newborn care, which includes administration of antibiotics, treatment for eclampsia and preeclampsia, treatment of postpartum hemorrhage, manual removal of the placenta, assisted vaginal delivery (vacuum extraction), removal of retained products of conception, and neonatal resuscitation. [(33)](https://www.zotero.org/google-docs/?AVE9aw)

| **Service** | **Structural quality measures** | **Survey reference** |
| --- | --- | --- |
| Family planning | - Group IEC/BCC meetings held - Qualified staff trained in family planning - Penis model available - Products in stock - Family planning patient register - National protocol for reproductive health and family planning - IUD insertion equipment: speculum, dilator, gloves, scissors | Q 3.24  Q 3.66  Q 3.69  Q 11.20 30-34  Q 12.24  Q 14.58 66  Q 1509 ak, ao, o, j |
| Antenatal care | - Conduct outreach for antenatal care - Iron and folate routinely prescribed - Mebendazole routinely prescribed - Qualified person provides care - Functional weighing scale - Tetanus toxoid vaccine in stock - Iron tabs in stock - Folate in stock - Pregnancy tests in stock - ANC register - Antenatal care national standards - Functional fetoscope | Q 3.25  Q 3.26  Q 3.27  Q 3.30c  Q 3.31  Q 11.20 59  Q 11.20 10  Q 11.20 11  Q 11.20 68  Q 12.25  Q 14.58 68  Q 15.02 j |
| Spontaneous childbirth  (BEmONC) | - Partograph forms available - Staff trained in vacuum extractor use - Capability to administer antibiotics, oxytocin, and anticonvulsants - Capability to remove retained products - Availability of sterile gloves - Availability of sterilized obstetrical boxes - Availability of episiotomy boxes - Emergency obstetric drugs in stock - Delivery register - Newborn care national standards - Available ringer lactate & colloids - Available relevant equipment (aspirator, dilator, etc.) - Sufficient water and soap - Functional light in delivery room - Delivery room structures in good shape - Appropriate curtains - Delivery table in good condition - Delivery room is clean | Q 3.36  Q 3.39  Q 3.40  Q 3.41/3.42  Q 3.43  Q 3.44  Q 3.45  Q 11.20 51-56  Q 12.26  Q 14.58 69  Q 15.02 w, x  Q 15.09 a-as  Q 14.23  Q 14.24  Q 14.25/14.27  Q 14.26/14.28  Q 14.30  Q 14.29 |
| Childhood immunization | - Vaccines in stock - National protocol for child vaccination - Available vaccine thermometer - Available cold box / carrier - Available refrigerator | Q 11.20 57-65 (excl. 59)  Q 14.58 65  Q 15.06 a  Q 15.06 b  Q 15.06 d |
| Malaria | - Treatments in stock - Diagnostic RDTs in stock - Able to perform diagnostic in last 3 months - National protocol for malaria diagnosis and treatment | Q 11.20 35-39  Q 11.20 66  Q 10.08_test 04  Q 14.58 64 |
| Tuberculosis | - Separate room for tuberculosis patients - Tuberculosis register - Able to perform tuberculosis smear - Laboratory TB register - Diagnostic kits in stock - Treatments in stock - National protocol for tuberculosis diagnosis and treatment | Q 3.56  Q 3.58  Q 1008_test 05  Q 10.13  Q 11.20 50  Q 11.20 44-49  Q 14.58 61 |
| HIV | - Qualified staff trained as HIV counselor - All counseling done by trained staff - Private HIV counseling room - Able to perform HIV testing in last 3 months - Able to test HIV viral load in last 3 months - Able to test CD4+ count in last 3 months - HIV/AIDS VCT & PMTCT services register - HIV treatment and referral register - Diagnostic tests in stock - National HIV testing and counseling guidelines | Q 3.75  Q 3.76  Q 3.77  Q 10.08_test 07  Q 10.08_test 08  Q 10.08_test 09  Q 12.28  Q 12.29  Q 11.20 67  Q 14.58 73 |
| Other STIs | - Able to test syphilis (RPR) in last 3 months - Able to do urine dipstick in last 3 months - Diagnostics in stock - Management of Sexually Transmitted Infections (STI) guidelines | Q 10.08_test 12  Q 10.08_test 13  Q 11.20 69 & 70  Q 14.58 72 |

Table A1.4. Additional details from results reported in Table 1.

| **Measure** | **Std dev, Control** | **Std dev, DFF** | **Std dev, PBF** | **% zero, Control** | **% zero, DFF** | **% zero, PBF** | **U-value, C v D** | **U-value, C v P** | **U-value, D v P** |
| --- | --- | --- | --- | --- | --- | --- | --- | --- | --- |
| # Service offered, total | 6.8 | 8.5 | 4.5 | 3% | 6% | 0% | 38,761 | 28,412 | 41,744 |
| # Lab diagnostics, 3 mo. | 4.3 | 4.3 | 3.9 | 45% | 26% | 9% | 33,390 | 25,930 | 42,682 |
| # POC diagnostics, today | 1.5 | 1.7 | 1.3 | 25% | 12% | 2% | 24,899 | 15,714 | 45,354 |
| # General drugs, today | 5.9 | 6.9 | 4.3 | 17% | 9% | 0% | 25,318 | 12,724 | 41,667 |
| # FP options offered | 2.1 | 1.7 | 1.1 | 27% | 16% | 1% | 42,277 | 39,262 | 44,180 |
| # Pregnancy services | 1.4 | 1.7 | 1 | 5% | 12% | 2% | 38,004 | 29,566 | 45,721 |
| # immunizations offered | 1.9 | 2.8 | 1 | 3% | 8% | 0% | 48,395 | 50,024 | 52,750 |
| # malaria services | 1 | 1 | 0.8 | 18% | 14% | 3% | 33,390 | 33,206 | 56,364 |
| # HIV + STI services | 1.6 | 1.7 | 1.8 | 48% | 40% | 16% | 38,509 | 31,870 | 44,992 |

Table A1.5. Regression results looking at the effect size of intervention and available revenues on facility service offerings. More detailed descriptions of the measures are provided in Table S1.1. DFF = direct facility financing. PBF = performance-based financing. POC = point of care. FP= family planning. HIV = human immunodeficiency virus. STI = sexually transmitted infections. Statistical significance is indicated by *, where *** is p < 0.001, ** is p < 0.01, and * is p < 0.05.

| **Measure** | **R Sq.** | **Co-efficient Revenue** | **Co-efficient DFF** | **Co-efficient PBF** |
| --- | --- | --- | --- | --- |
| # Service offered, total | 0.26 | 4.92 *** | 5.62 *** | 6.56 *** |
| # Lab diagnostics, 3 mo. | 0.28 | 1.91 *** | 1.61 *** | 2.34 *** |
| # POC diagnostics, today | 0.28 | 0.71 *** | 1.91 *** | 2.12 *** |
| # General drugs, today | 0.34 | 2.65 *** | 8.55 *** | 10.07 *** |
| # FP options offered | 0.25 | 0.57 ** | 1.46 *** | 1.88 *** |
| # Pregnancy services | 0.15 | 0.54 *** | 0.90 *** | 1.25 *** |
| # immunizations offered | 0.05 | 0.53 * | (0.23) | 0.66 |
| # malaria services | 0.25 | 0.84 *** | 1.27 *** | 1.02 *** |
| # HIV + STI services | 0.25 | 1.52 *** | Not significant | Not significant |
